# Mild behavioral impairment and risk of incident dementia: A systematic review and meta-analysis

**DOI:** 10.1177/13872877261480315

**Published:** 2026-09-07

**Authors:** Karina L. M. Sassi, Anika Thomson, Vanessa M. Young, Natalia P. Rocha, Antonio L. Teixeira

**Affiliations:** 1 Department of Neurology, 12340 University of Texas Health Science Center at Houston, Houston, TX, USA; 2 Biggs Institute for Alzheimer's & Neurodegenerative Diseases, 14742 University of Texas Health Science Center at San Antonio, San Antonio, TX, USA

**Keywords:** Alzheimer's disease, amyloid, dementia, early diagnosis, neurodegeneration

## Abstract

**Background:**

Late-life emergence of persistent behavioral changes has been recognized as a clinically meaningful sign of neurodegeneration. These neuropsychiatric symptoms, operationalized as mild behavioral impairment (MBI), represent a unique pathway through which early dementia symptoms may become observable. By capturing this neurobehavioral dimension of risk, MBI expands the traditional focus on cognitive decline and may provide a complementary framework for understanding the early manifestations of neurodegeneration.

**Objective:**

This systematic review evaluated longitudinal evidence on the association between MBI and incident dementia, including conversion rates, dementia subtypes, domain-specific risk patterns, and biomarker correlates.

**Methods:**

We systematically searched five databases up to November 2025. Eligible studies were longitudinal prospective or retrospective cohorts including adults ≥ 50 years without dementia at baseline, assessing MBI using ISTAART-aligned instruments and reporting incident dementia outcomes. Two reviewers independently screened studies and extracted data. Hazard ratios were synthesized using random-effects meta-analysis, with heterogeneity and publication bias formally assessed.

**Results:**

Despite significant study heterogeneity, MBI was associated with increased risk of incident dementia. Alzheimer's disease was the most frequently reported outcome. Among different neurobehavioral domains, apathy and affective dysregulation emerged as particularly strong predictors of dementia risk. A few biomarker studies linked MBI to greater amyloid burden, elevated plasma phosphorylated tau, and structural brain changes, supporting a biological connection between MBI and early neurodegeneration.

**Conclusions:**

MBI seems to represent a clinically meaningful syndrome that identifies individuals at elevated risk for dementia. Future studies must confirm the clinical and epidemiological value of MBI and/or its subdomains, exploring their biological substrates.

## Introduction

Neuropsychiatric symptoms (NPS) have long been recognized as common features across dementias.^1^ However, for many years they were conceptualized primarily as manifestations of an established disease rather than as potential early indicators of its onset.^2^ Late-life changes in mood, motivation, personality, and/or impulse control were often attributed to psychosocial stressors, physical comorbidities, or primary psychiatric disorders, and consequently received limited attention in the context of dementia.^3^ In addition, psychiatric disorders, especially depression, have been considered risk factors of dementia.^4,5^ As the evidence from longitudinal cohort studies accumulated, it became increasingly clear that NPS frequently emerge years before the onset of measurable cognitive impairment, suggesting that behavioral disorders may represent an early clinical expression of underlying pathology.^6^

In response to the need for a framework to characterize late-life emergent behavioral changes, the International Society to Advance Alzheimer's Research and Treatment (ISTAART) Neuropsychiatric Syndromes Professional Interest Area proposed the construct of Mild Behavioral Impairment (MBI).^7^ Introduced in 2016, MBI established standardized criteria for identifying and categorizing new-onset, persistent NPS in older adults as a possible prodromal stage of dementia. The criteria emphasized later-life onset, persistence for at least six months, and a clear deviation from longstanding patterns of behavior. These features distinguish MBI from chronic psychiatric disorders and transient emotional reactions.^8^

The MBI framework was developed to address several gaps in early identification of dementia. First, commonly used cognitive screening tools, such as the Mini-Mental State Examination (MMSE), are brief and user-friendly but demonstrate limited sensitivity to detect early Alzheimer's disease (AD) or other dementias. They also have difficulty distinguishing mild cognitive impairment (MCI) from healthy aging and tend to overlook non-cognitive symptom domains. Although alternative instruments are available, their sensitivity, specificity, and suitability vary across populations, and no single test is ideal for all dementias.^9^ Second, the lack of standardized criteria for late-life behavioral changes meant that the prognostic significance of emergent psychiatric symptoms was often overlooked or misattributed to primary psychiatric illness, leading to systematic under recognition of their potential as early indicators of dementia.^10^ Third, emerging biomarker research has suggested that NPS may correspond to underlying pathological processes, including amyloid deposition and/or tau abnormalities, thereby underscoring the biological relevance of behavioral changes in the prodromal stages of dementia.^11^

As disease-modifying treatments begin to enter clinical practice, the capacity to detect neurodegenerative processes before cognitive symptoms emerge becomes increasingly relevant. In this context, MBI represents a promising behavioral marker that may help identify individuals in the earliest, most treatment-responsive stages of disease.

Since its introduction, several groups have examined MBI in longitudinal cohorts, evaluating whether individuals who meet MBI criteria are at increased risk of cognitive decline and incident dementia. Although these studies vary in methodology, sample size, and clinical setting, they collectively contribute to a growing body of evidence linking MBI to dementia. The current review synthesizes longitudinal findings from studies that applied ISTAART-consistent MBI criteria or operationalized definitions derived from them, with particular attention to conversion rates, domain-specific predictors, dementia subtypes, and biomarker correlates. The aim is to provide an updated assessment of the extent to which MBI may function as an early behavioral indicator of dementia and to propose an integrated framework that bridges its clinical and biological dimensions, highlighting both research and practical implications.

## Methods

### Literature search

We conducted a systematic review of studies examining the longitudinal association between MBI and the subsequent development of dementia among individuals without dementia at baseline. Our search covered five electronic databases up to November 9, 2025: PubMed, Embase, Scopus, PsycInfo, and Scielo. In addition, a manual search identified one additional study. This systematic review adheres to the Preferred Reporting Items for Systematic Reviews and Meta-Analyses (PRISMA) guidelines^12^ and is registered with Open Science Framework (OSF)^13^ as an OSF Preregistration (https://osf.io/qkm57).

### Search strategy and selection

The search strategy was customized for each database and is described in Supplemental Table 1. Two researchers (K.L.M.S. and A.T.) independently reviewed the articles to ensure an unbiased selection process based on predefined eligibility criteria. Any disagreements were resolved through discussions with additional authors. The eligibility criteria followed the PICO (Population, Intervention, Comparison, Outcome)^14^ framework, ensuring that the selected studies aligned with the objectives of the review.

### Inclusion criteria

Studies were eligible for inclusion if they employed a longitudinal design, including either prospective or retrospective cohort methodologies with clearly defined baseline assessments. The population of interest comprised adults aged 50 years or older who did not have dementia at baseline. Eligible studies were required to assess MBI according to the ISTAART criteria using validated instruments such as the MBI Checklist, Neuropsychiatric Inventory, or other validated instruments that could be operationalized to establish an MBI diagnosis consistent with ISTAART guidelines.^8^ The primary outcome was incident dementia, whether all-cause or specific subtypes, diagnosed using standardized frameworks such as DSM-5^15^ and Clinical Dementia Rating (CDR) scores.^16^ No restrictions were placed on year of publication, follow-up duration, but only peer-reviewed articles were considered. Because our research group includes multilingual members and has access to several validated translated tools, we decided not to apply language restrictions, thereby reducing potential selection bias.

### Exclusion criteria

Studies were excluded if they involved animals, employed a cross-sectional design, or were case reports, narrative reviews, systematic reviews, editorials, commentaries, or other publication types that did not align with the objectives of this review. We also excluded studies that did not use a standardized method to assess MBI or that lacked a clearly defined dementia outcome. Additionally, studies including participants who already had dementia at baseline were considered ineligible.

Studies were excluded for any of the following reasons: (1) the population was not of interest (e.g., study examining hearing loss and MBI without assessing dementia incidence); (2) the outcome was not of interest (e.g., focus on sleep quality and MBI without evaluating cognitive or dementia-related outcomes); (3) the exposure was not of interest (e.g., studies assessing behavioral symptoms using criteria other than ISTAART-defined MBI); (4) the study design was ineligible (e.g., reviews, editorials, or other non-original research articles); or (5) the publication was limited to a conference abstract without full data available.

### Meta-analysis methodology

The meta-analysis focused on studies reporting hazard ratios (HRs) for the association between MBI and incident dementia. Because HRs incorporate a temporal component and were the most consistently reported effect measure, studies reporting odds ratios (ORs) were excluded to avoid conceptual and statistical incompatibility.^17,18^ All analyses were conducted in R version 4.5.2 using the ‘*metafor’* package (version 4.8-0).^19,20^

In accordance with Cochrane recommendations, HRs and their 95% confidence intervals were transformed to the natural logarithmic scale,^21^ and standard errors were calculated from the reported confidence intervals. A random-effects model was used to account for anticipated heterogeneity across study populations, instruments, and follow-up durations.

Eight studies met the inclusion criteria.^22–29^ One study had two independent HRs due to stratification by traumatic brain injury history.^27^ For Mudalige et al., only the estimate derived from participants without sleep disturbance was included to minimize potential confounding.^26^ Studies evaluating specific MBI domains (e.g., apathy, affective dysregulation) were retained when domain-level measures constituted the primary operationalization of MBI.^22,29^ Sun et al., which restricted outcomes to MCI or AD, was included despite its narrower outcome definition to ensure comprehensive coverage.^28^

Statistical heterogeneity was quantified using the I^2^ statistic. Publication bias was evaluated through visual inspection of funnel plot and Egger's regression test.

**Figure 1.**
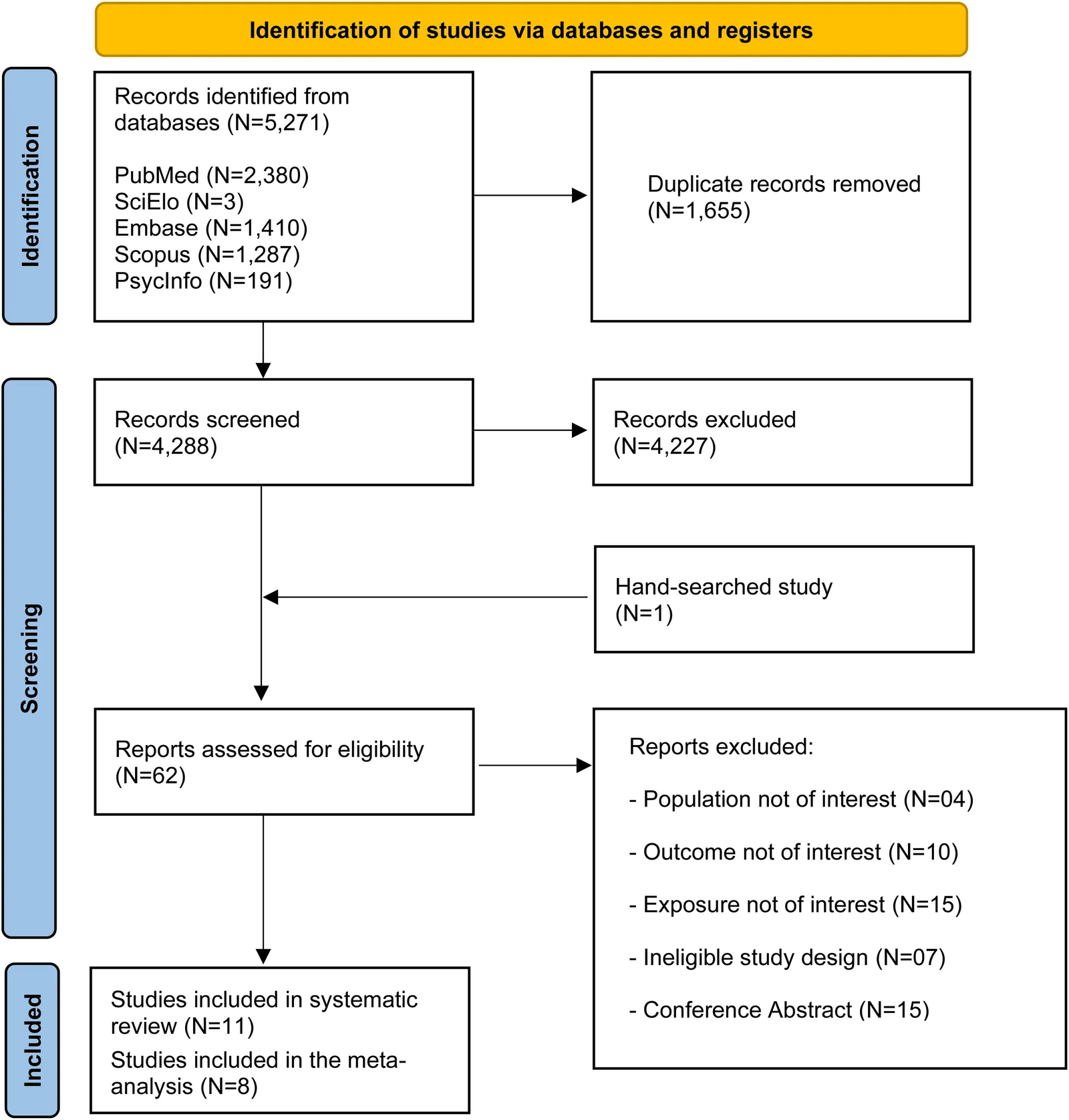
PRISMA flow diagram. Page MJ, McKenzie JE, Bossuyt PM, et al. The PRISMA 2020 statement: an updated guideline for reporting systematic reviews. *BMJ* 2021; 372: n71.

## Results

The study selection process is illustrated in Figure 1. A total of 5271 records were identified across five databases. Duplicates (n = 1, 655) were identified and removed using reference management software (Rayyan)^30^ which automatically flagged repeated records. This process was further verified through manual review. After duplicate removal, title and abstract screening resulted in the exclusion of 4227 articles.

**Figure 2.**
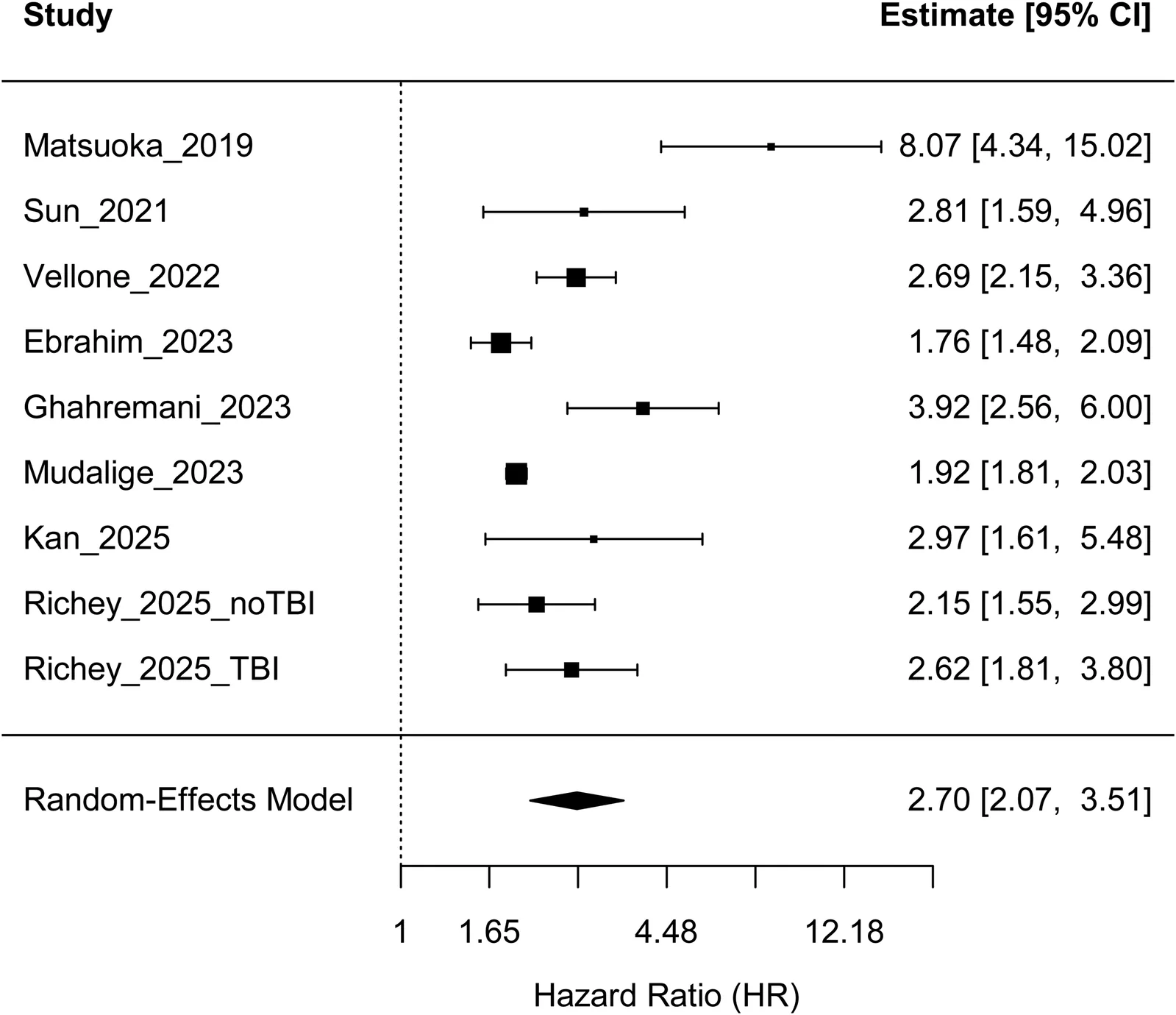
Forest plot of the studies included in the meta-analysis.

Subsequently, 61 full-text articles were assessed for eligibility according to predefined inclusion and exclusion criteria, with one additional study identified through manual reference screening. Of the 62 articles evaluated at full-text, 11 met the inclusion criteria and were included in the final synthesis (Table 1). Eight studies were included in the meta-analysis. A comprehensive list of excluded studies and the reasons for exclusion at the full-text stage are provided in Supplemental Table 2. The risk of bias, as assessed by the Newcastle-Ottawa scale (Supplemental Table 3), was considered low for all studies.

**Table 1.** Characteristics of the studies included in the systematic review.

Author, year / country	Objective	Design of study	Sample size and characteristics	Age/Mean age (years) ± SD	Instrument tools	Main results
Matsuoka et al., 2019 ^25^ Japan	To assess the prevalence of MBI, and compare the incidence of dementia in MBI with that in other psychiatric diseases	Retrospective observational cohort	N = 2853 dementia-free participants (only 1329 followed up for at least 1 month) Source Population: Psychiatric outpatient clinic Female: 62.3%	68.9 ± 11.1	Clinical Assessment for RBD DLB consensus criteria ICD-10 MBI was retrospectively diagnosed using ISTAART research diagnostic criteria Petersen's criteria (MCI)	The prevalence of MBI was 3.5%; Total incidence of dementia: 5.8% (77/1 329); Among patients who developed dementia, the most frequent baseline diagnosis was MBI, accounting for 29 cases (37.7%), followed by MCI without MBI (17 cases, 22.1%); The most common type: AD (85.7%); One patient was diagnosed with DLB, among the three patients with prior RBD.
Orso et al., 2020 ^31^ Italy	To identify baseline clinical and MRI features associated with conversion to neurodegenerative disease, and to validate practical criteria for identifying MBI individuals at higher dementia risk	Retrospective observational cohort	N = 113 MBI patients were randomly assigned to Group A (n = 56) or Group B (n = 57) Source Population: Behavioral neurology/adult psychiatric services Female: 28.3%	Group A: 65.4 ± 7.9 Group B: 66.6 ± 6.4	Letter Fluence Test MMSE MRI NPI Rey-Auditory Verbal Learning Test Rey Figure Recall and Auditory Verbal Learning Test RMET TMT A TMT B	Group A: 14 converters to bvFTD and 4 converters to AD. Converters showed lower executive function scores, lower RMET scores, higher NPI scores, and presented more severe focal frontal atrophy at baseline; Group B: 13 converters to bvFTD; and none to AD. Combination of variables identified in Group A significantly discriminated converters from nonconverters.
Ismail et al., 2021 ^18^ Canada	To assess the associations of MBI and SCD, alone and in combination, with incident cognitive and functional decline in cognitively normal older adults	Retrospective observational cohort	N = 2769 (1536 MBI−SCD−; 254 MBI−SCD+; 743 MBI + SCD−; 236 MBI + SCD+) Source Population: NACC database Female: 63.3%	73.0 ± 9.2	CDR NPI-Q SCD-Initiative Workgroup criteria	MBI + SCD−: Dementia incidence 2.7%; highest risk vs. reference (OR≈21.2; AOR≈19.2); MBI + SCD+: Dementia incidence 1.3%; increased risk vs. reference, lower than MBI + SCD− (OR≈9.9; AOR≈9.3); MBI−SCD+: Dementia incidence 0.4%; low risk, similar to reference group; MBI−SCD− (reference): Dementia incidence 0.1%.
Sun et al., 2021 ^28^ China	To examine the roles of AD imaging pathologies in modulating the associations of MBI with cognitive impairments	Retrospective observational cohort	N = 1129 (744 No MBI; 385 MBI) Source Population: ADNI Female: 49.9%	No-MBI: 71.7 ± 7.3 MBI: 72.4 ± 6.3	Amyloid PET for AD biomarkers Cognitive section of ADAS MMSE MoCA NPI-Q	MBI predicted faster increase in brain amyloid and higher risk of clinical conversion to AD (HR = 2.42); MBI associated with worse global cognition and higher Aβ burden; Aβ partially mediated MBI effects on cognition (mediation 14.67–40.86%); No significant association between MBI and tau burden or neurodegeneration;
Vellone at al., 2022 ^29^ Canada	To examine the relation between MBI-related apathy and dementia progression, and to explore how sex, race, cognitive status, and *APOE* genotype may influence this relation	Retrospective observational cohort	N = 3932 (3578 No-NPS; 354 MBI apathy) Source Population: NACC Female: 60.1%	No-NPS: 72.9 ± 9.8 MBI apathy: 76.2 ± 8.8	*APOE* genotype NPI-Q	MBI-apathy vs. no-NPS: MBI-apathy had higher progression to dementia (39.8 vs. 7.4%); *APOE* ɛ4 increased risk, ɛ2 decreased risk for dementia; Of the MBI participants who progressed to dementia, 80.9% developed AD, 4.3% bv-FTD, 5.0% LBD, and 2.8% VaD; MBI-apathy vs. no-apathy: contribution of apathy to dementia was greater in NC than in MCI (HR: 5.91 vs. 2.16).
Ebrahim et al., 2023 ^22^ Canada	To evaluate the long-term connection between MBI-affective dysregulation with incident dementia	Retrospective observational cohort	N = 4984 (3698 no-NPS; 1 286 MBI-affective dysregulation) Source Population: NACC Female: 60.6%	No-NPS: 72.8 ± 9.8 MBI-affective dysregulation: 75 ± 9.6	*APOE* ε4 status NPI-Q	MBI-affective symptoms increased dementia risk 1.76-fold (higher in Black than White participants); MBI-affective dysregulation: 25.3% developed dementia: 85.5% AD, 3.7% DLB, 2.5% bvFTD, 1.2% VaD, 6.8% unrecorded; No NPS: 7.6% developed dementia: 89.3% AD, 1.1% DLB, 1.1% bvFTD, 2.1% VaD, 6.4% unrecorded; MBI–affective dysregulation increased dementia risk (NC: HR 2.59; MCI: HR 1.50). Effect was stronger in *APOE* ε4 noncarriers (HR 2.11) than carriers (HR 1.44).
Ghahremani et al., 2023 ^23^ Canada	To assess the link between MBI and p-tau181 levels, neuropsychological test performance and incident AD	Retrospective observational cohort	N = 571 (103 MBI; 135 NPS-not-MBI; 333 No NPS / 201 CN; 370 MCI) Source Population: ADNI Female: 46.8%	MBI: 72.1 ± 7.25 NPS-not-MBI: 72.2 ± 7.14 No NPS: 72.2 ± 7.03	MMSE NPI/NPI-Q Plasma p-tau181 RAVLT TMT B	MBI vs. no NPS: MBI had higher baseline plasma p-tau181 and greater longitudinal increase in p-tau181 over 4 years; MBI vs. no NPS: greater decline in memory and executive function over 4 years; higher risk of incident dementia over 5 years (HR = 3.92); NPS-not-MBI vs. no NPS: no significant differences in p-tau181, cognitive decline, or dementia risk; Among p-tau181 positive participants, MBI associated with increased dementia incidence (HR = 2.56); All incident cases developed AD.
Mudalige et al., 2023 ^26^ Canada	To investigated the longitudinal associations between MBI, Sleep Disturbances, and incident dementia.	Retrospective observational cohort	1) Baseline MBI → incident Sleep Disturbance: N = 11 277 (59% Female) 2) Baseline Sleep Disturbance → incident MBI: N = 10 535 (60.9% Female) 3) MBI within sleep-disturbed older adults → incident dementia: N = 13 544 (50.1% Female) 4) MBI within non-sleep-disturbed older adults → incident dementia: N = 11 921 (58.6% Female) Source Population: NACC	Analysis 1: 72.7 ± 8.8 Analysis 2: 72.7 ± 8.8 Analysis 3: 72.2 ± 8.8 Analysis 4: 72.9 ± 8.8	NPI-Q	Baseline MBI predicted 3.1× higher risk of developing sleep disturbance; Baseline sleep disturbance predicted 1.5× higher risk of developing MBI; Co-occurrence of MBI and sleep disturbance predicted a 2.2× greater risk of dementia compared to sleep disturbance alone; They did not differentiate between types of dementia.
Gracia-García et al., 2024 ^17^ Spain	To examine how MBI and its specific domains relate to the risk of developing dementia among older adults	Retrospective observational cohort	N = 3 947 (3 526 CN; 421 CI-ND) Source Population: Community-dwelling individuals (ZARADEMP project) Female: 53.3%	72.8 ± 9.5	AGECAT bADL EURODEM risk factors questionnaire GMS-B iADL HAS MMSE	MBI more frequent in CI-ND (56.8%) than CN (37.2%); CN with MBI had higher all-cause dementia risk (7.0% vs. 4.6%); CN individuals with MBI had a similar AD conversion rate to those without MBI (3.6%); Decreased motivation predicted all-cause dementia after adjustment; Overall MBI predicted dementia in bivariate but not multivariable analysis.
Kan et al., 2025 ^24^ Singapore	To examine the association between MBI and MRI measures reflecting comorbid cerebrovascular disease and neurodegeneration, and to investigate how MBI and MRI burden interact to influence cognitive and clinical trajectories in dementia-free older adults	Longitudinal prospective study	N = 253 dementia-free participants; 14.6% with MBI; Source Population: two memory clinics in Singapore Female: 51.8%	71.9 ± 7.7 years	Animal Fluency Color Trails Tests 1 and 2 CDR Digit Span Test Hopkins Verbal Learning Test Modified Boston Naming Test MRI NPI Rey-Osterrieth Complex Figure Test SDMT	Lower composite MRI scores (greater mixed CeVD + neurodegeneration) were associated with higher likelihood of MBI (OR = 3.88); WMH volume and reduced cortical thickness were individually associated with MBI; MBI participants showed greater clinical (CDR) and cognitive decline over time; The strongest decline occurred in participants with MBI and low MRI scores (greater pathology burden); 43.2% of participants with MBI vs. 15.3% in the non-MBI group developed dementia.
Richey et al., 2025 ^27^ United States	To investigate if cross-sectional associations of cognitive status with MBI symptoms differ by TBI status and if prospective associations of MBI domain positivity with incident dementia risk differs by TBI status.	Retrospective observational cohort	N = 2 246 Source Population: Community based cohort Female: 59%	75.6 ± SD not reported	Eight-item Informant Interview Hospital/death certificate codes Six-Item Screener FAQ MMSE NPI-Q Self-report TBI	MCI associated with higher odds of MBI, independent of TBI status (with TBI: OR = 2.04; without TBI: OR = 1.60); Presence of ≥1 MBI domain predicted higher risk of incident dementia, regardless of TBI (no TBI: HR = 2.15; with TBI: HR = 2.62).

AD: Alzheimer's disease; ADAS: Alzheimer's Disease Assessment Scale; ADNI: Alzheimer's Disease Neuroimaging Initiative; AGECAT: Automated Geriatric Examination for Computer Assisted Taxonomy; AOR: adjusted odds ratio; *APOE*: apolipoprotein E; Aβ: amyloid-β; bADL: basic activities of daily living; bvFTD: behavioral variant frontotemporal dementia; CDR: Clinical Dementia Rating; CeVD: cerebrovascular disease; CI-ND: cognitive impairment–no dementia; CN: cognitively normal; DLB: dementia with Lewy bodies; EURODEM: European Studies of Dementia; FAQ: Functional Activities Questionnaire; GMS-B: Geriatric Mental State B; HAS: History and Etiology Schedule; HR: hazards ratio; iADL: instrumental activities of daily living; ICD: International Classification of Diseases; ISTAART: Alzheimer's Association International Society to Advance Alzheimer's Research and Treatment; LBD: Lewy body dementia; MBI: mild behavioral impairment; MCI: mild cognitive impairment; MMSE: Mini-Mental State Examination; MoCA: Montreal Cognitive Assessment; MRI: magnetic resonance imaging; NACC: National Alzheimer's Coordinating Center; NC: normal cognition; NPI: Neuropsychiatric Inventory; NPI-Q: Neuropsychiatric Inventory Questionnaire; NPS: neuropsychiatric symptoms; OR: odds ratio; PET: positron emission tomography; RAVLT: Rey Auditory Verbal Learning Test; RBD: REM sleep behavior disorder; RMET: Reading the Mind in the Eyes Test; SCD: subjective cognitive decline; SDMT: Symbol Digit Modalities Test; TBI: traumatic brain injury; TMT: Trail Making Test; VaD: vascular dementia; WMH: white matter hyperintensity.

### General characteristics

The included studies comprised predominantly observational cohort studies, most of which employed retrospective designs,^17,18,22,23,25–29,31^ with one prospective longitudinal study.^24^ Study populations were drawn from diverse clinical and community settings, including a psychiatric outpatient clinic,^25^ a behavioral neurology/adult psychiatric services,^31^ a memory clinic,^24^ a community-dwelling cohort,^27^ and large multicenter databases.^17,18,22,23,25,26,28,29^ Geographically, studies were conducted across Asia (Japan, China, Singapore),^24,25,28^ Europe (Italy, Spain),^17,31^ and North America (Canada and the United States),^18,22,23,26,27,29^ with a strong representation of Canadian studies^18,22,23,26,29^ largely based on the National Alzheimer's Coordinating Center (NACC) database.^18,22,26,29^ Sample sizes varied substantially, ranging from 113 participants in a single-center Italian cohort^31^ to over 13,000 individuals in large population-based analyses.^26^

MBI was most operationalized using informant-based neuropsychiatric assessments, particularly the Neuropsychiatric Inventory (NPI)^23,24,31^ and/or the Neuropsychiatric Inventory Questionnaire (NPI-Q)^18,22,23,26–29^ through algorithms aligned with the ISTAART criteria. One study reported that MBI was retrospectively diagnosed using the ISTAART research diagnostic criteria without specifying the use of the NPI-Q,^25^ while another study used the Geriatric Mental State-B.^17^ Cognitive status was also assessed using a variety of scales, as shown in Table 1. Several studies incorporated biomarkers, including amyloid PET imaging,^28^ plasma phosphorylated tau at threonine 181 (p-tau181),^23^ *APOE* genotyping,^22,29^ and structural MRI markers of neurodegeneration and cerebrovascular disease.^24,31^

### MBI prevalence and baseline characteristics

All studies comprised participants without dementia at baseline, in accordance with the inclusion criteria. MBI prevalence among dementia-free individuals varied substantially, ranging from 3.5%^25^ to 39.3%.^17^ The participants were predominantly older adults, with mean ages consistently falling in the early to mid-70 s across most cohorts.^17,18,22–24,26–29^ Younger mean ages were observed in clinic-based^31^ or psychiatric clinic samples,^25^ where participants were generally in their mid to late 60 s. Sex distribution also varied across cohorts but generally reflected a small female predominance.^17,18,22,24–27,29^

Baseline cognitive status was not systematically assessed using a single classification framework. Several cohorts included both cognitively normal individuals and those with MCI.^17,22–25,27–29^ One study also included subjective cognitive decline,^18^ which was not assessed or reported in the remaining studies. One study included only participants with MBI at baseline^31^ while another did not evaluate cognition at baseline.^26^

### Conversion rates to dementia

Conversion to dementia was evaluated, with different follow-up duration and outcome definitions. As a result, reported conversion rates differed depending on study population, MBI operationalization, and baseline cognitive status.

In a psychiatric outpatient cohort from Japan,^25^ the overall incidence of dementia among dementia-free participants was 5.8%. Although the study required only a minimum of one month of follow-up after the baseline MBI assessment, the authors did not report the median or total duration of follow-up. Among those who converted, MBI was the most frequent baseline diagnosis, accounting for 37.7% of incident cases, surpassing conversion proportion observed in individuals with MCI without MBI (22.1%). Similarly, a clinic-based Italian cohort,^31^ in which all participants met MBI criteria at baseline, reported a 27.4% conversion rate to dementia over four years.

Evidence from the NACC database further highlighted the prognostic relevance of MBI. One study^18^ found that, over a three-year period, dementia incidence was highest among individuals with MBI without subjective cognitive decline (2.7%), followed by those with both MBI and subjective cognitive decline (1.3%). In contrast, individuals without MBI exhibited substantially lower conversion rates regardless of subjective cognitive decline status, with those without MBI but with subjective cognitive decline showing a 0.4% incidence, and individuals without MBI and without subjective cognitive decline showing 0.1% over the same period.

Findings from Alzheimer's Disease Neuroimaging Initiative (ADNI) studies reinforced these associations. One study^28^ reported that individuals with MBI had a significantly higher risk of conversion to AD (HR = 2.42) compared with those without MBI. Another ADNI report^23^ similarly found an elevated five-year risk of incident dementia among participants with MBI (HR = 3.92) compared with those who had NPS without MBI and those without NPS.

Additional cohort analyses provided complementary insights. A large NACC-based study^26^ demonstrated that the co-occurrence of MBI and sleep disturbance conferred a greater risk of incident dementia than sleep disturbance alone. The rate of developing MBI was 1.5-fold higher in older adults with baseline sleep disturbance compared with those without, and the risk of developing dementia was more than twice (2.2-fold) as high in individuals with both MBI and sleep disturbance relative to those with sleep disturbance alone. A community-based Spanish cohort^17^ observed that, among cognitively normal individuals, MBI domain of decreased motivation was associated with a higher risk of all-cause dementia, with affected individuals showing more than twice the odds of conversion (OR: 2.30) in multivariable analyses compared with those without MBI.

More recently, a study^24^ reported that 43.2% of individuals with MBI developed dementia over a mean follow-up of 49.89 months, compared with 15.3% of those without MBI, especially among participants with greater combined cerebrovascular and neurodegenerative MRI burden. MRI burden was derived from a weighted composite of white matter hyperintensity volume, infarct presence, hippocampal volume, and cortical thickness in AD-related regions, with lower scores reflecting greater burden. Finally, a U.S. community-based cohort^27^ showed that the presence of one or more MBI domains predicted a higher risk of incident dementia over a median follow-up of 6.4 years, with individuals endorsing ≥1 MBI domains exhibiting more than double the risk of conversion (HR = 2.15–2.62), independent of traumatic brain injury history.

### Dementia subtypes following MBI

Across studies that reported dementia subtypes, AD was the most frequently assigned clinical diagnosis among individuals with MBI, consistent with the predominance of AD (often mixed with vascular pathology) in population-based cohorts.^32^

One study published in 2019^25^ reported that, among 1329 psychiatric outpatients followed for an average of 22.7 months, 77 individuals developed dementia, the vast majority of whom were diagnosed clinically with AD (66/77; 85.7%). In this cohort, one patient developed dementia with Lewy bodies among the three individuals with a prior history of rapid eye movement sleep behavior disorder.

Findings from large registry-based cohorts further reinforced the predominance of AD following MBI. Using NACC data, two studies reported that over 80% of dementia cases among individuals with MBI-related apathy or affective dysregulation symptoms progressed to AD, while behavioral variant frontotemporal dementia, Lewy body dementia, and vascular dementia accounted for smaller proportions of cases.^22,29^ Consistent with these observations, a study based on the ADNI cohort reported that all incident dementia cases following MBI converted to AD.^23^ Over a five-year follow-up, 70 participants (12.3%) developed dementia, and all cases were classified as AD. Although the study did not report how many of these conversions occurred specifically within the MBI group, the survival analyses showed that individuals with MBI had the lowest dementia-free survival.

In contrast, a cohort derived from a behavioral neurology and adult psychiatric service reported that individuals with MBI most frequently converted to the behavioral variant of frontotemporal dementia, with comparatively fewer conversions to AD and no reported cases of other dementia subtypes.^31^ In this study, 27.4% of participants with MBI converted to dementia, of whom 87.1% were diagnosed with behavioral variant of frontotemporal dementia.

In NACC-based analyses examining the interaction between MBI and sleep disturbance, increased dementia risk associated with MBI was reported, but dementia subtypes were not differentiated.^26^ Another study based on the ZARADEMP project found that, within the cognitively normal group, the presence of MBI was associated with a higher risk of all-cause dementia compared with those without MBI (7.0% veresus 4.6%), but not AD.^17^ Other two studies^24,27^ evaluated incident dementia in community-based cohorts without providing detailed subtype classifications. One retrospective study analyzed only patients who developed AD over the follow-up period, without assessing conversions to other dementia subtypes.^28^

### MBI domains as predictors of dementia risk

Decreased motivation (apathy) emerged as a particularly strong domain-specific predictor of dementia. In a registry-based NACC analysis, individuals with MBI-related apathy showed a dementia progression rate of 39.8%, compared with 7.4% among those without NPS. The association between apathy and dementia was stronger in cognitively normal individuals than in those with MCI (HR = 5.91 versus 2.16).^29^ Similarly, In the ZARADEMP cohort, after adjustment, decreased motivation remained independently associated with all-cause dementia, whereas overall MBI predicted dementia in bivariate but not multivariable analysis.^17^

Affective dysregulation was also associated with increased dementia risk. In another longitudinal NACC-based study, 25.3% of individuals with MBI-affective dysregulation developed dementia, compared with 7.6% among those without NPS. The association between affective dysregulation and dementia was stronger in cognitively normal participants (HR = 2.59) than in those with mild cognitive impairment (HR = 1.50) and was more pronounced among *APOE* ε4 noncarriers (HR = 2.11 versus 1.44).^22^

Other studies investigated MBI in general, but did not stratify participants by specific MBI domains, limiting direct comparisons among domains.^18,23–28,31^

### Biomarkers and pathophysiological correlates

Evidence from biomarker studies supports a biological link between MBI and dementias. An ADNI-based cohort-based study^28^ found that MBI was associated with higher cerebral amyloid-β burden and worse global cognition, with amyloid pathology partially mediating the relationship between MBI and cognitive impairment. MBI was also associated with faster amyloid accumulation and an increased risk of clinical conversion to AD.

Plasma biomarkers further supported these associations. In a study by,^23^ individuals with MBI exhibited higher baseline plasma p-tau181 levels and showed greater longitudinal increases over time, which paralleled accelerated cognitive decline. Structural MRI analyses also revealed that MBI was linked to more pronounced declines in memory and executive function over a four-year period, as well as an increased risk of developing dementia over five years (HR 3.92).

Finally, one study^24^ reported that white matter hyperintensities and reduced cortical thickness were independently associated with MBI and that individuals with both MBI and greater comorbid cerebrovascular and neurodegeneration burden exhibited the steepest cognitive and clinical decline.

### Factors modifying the association between MBI and dementia risk

Several studies identified factors that modified the association between MBI and dementia risk. While *APOE* ε4 increased dementia risk in individuals with MBI-related apathy,^29^ it paradoxically reduced the relative risk of dementia associated with MBI-affective dysregulation compared to non-carriers.^22^ Sociodemographic factors such as race influenced outcomes, with stronger associations observed among Black participants in one NACC-based analysis.^22^ Comorbid conditions also shaped risk trajectories. One study^26^ demonstrated a bidirectional relationship between MBI and sleep disturbances, with their co-occurrence conferring a substantially higher risk of dementia than either condition alone. Another study^27^ found that the presence of one or more MBI domains predicted incident dementia irrespective of traumatic brain injury status.

### Meta-analysis results

Eight studies^22–29^ contributed nine independent effect sizes evaluating the association between MBI and incident dementia. The random-effects model yielded a statistically significant pooled estimate, with a combined HR of 2.70 (95% CI: 2.07–3.51), indicating that individuals with MBI had more than double the risk of developing dementia compared with those without MBI (Figure 2). All individual studies reported HRs greater than 1.0, indicating a consistently elevated risk across cohorts. The magnitude of effects ranged from 1.76 to 8.07, reflecting variability in study designs and measurement approaches.

Heterogeneity was substantial (I^2^ = 89.8%), indicating considerable variability in effect sizes across studies. Despite this, the overall association remained statistically robust. Visual inspection of the forest plot (Figure 2) showed all estimates aligned on the same side of the null, with larger studies clustering near the pooled effect.

**Figure 3.**
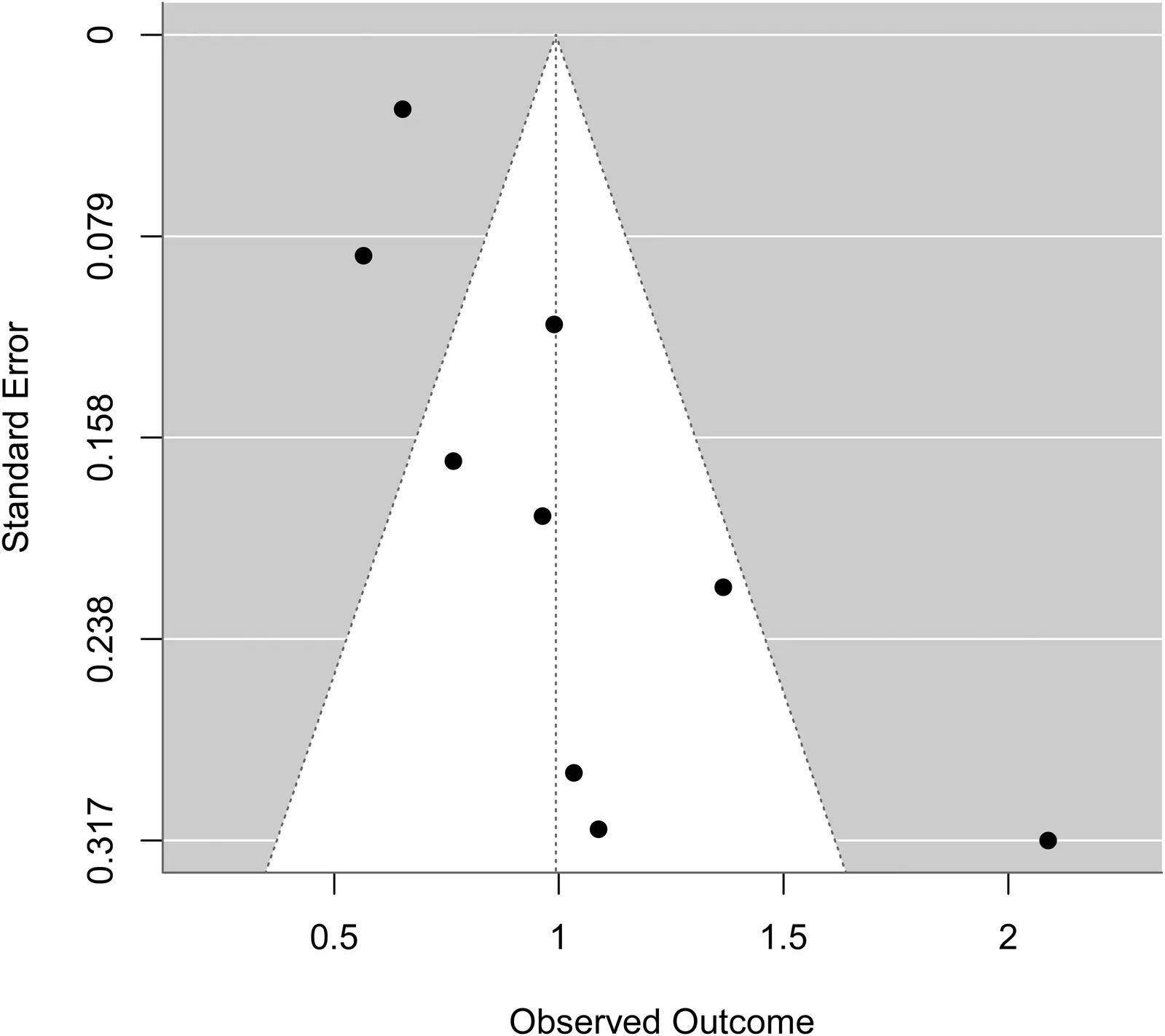
Funel plot assessing publication bias among the studies included in the meta-analysis.

Assessment of publication bias demonstrated funnel plot asymmetry (Figure 3), characterized by a relative absence of small studies reporting null or minimal effects. Egger's regression test confirmed this pattern (z = 3.41; p = 0.0006). One study contributed two stratified estimates,^27^ due to stratification by traumatic brain injury history, both of which were included independently in the pooled analysis.

## Discussion

The findings of this systematic review reinforce the conceptualization of MBI as an early clinical manifestation of dementia, offering both empirical and mechanistic insights into its prodromal stages. Across diverse populations and settings, MBI was consistently associated with elevated risk of conversion to dementia. Domain-specific analyses further highlighted the particularly strong predictive value of apathy and affective dysregulation.^17,22,29^ The robust association between apathy and incident dementia, especially among cognitively normal individuals,^29^ suggests that motivational deficits may reflect early disruptions within fronto-subcortical and limbic circuits. This interpretation aligns with neuropathological evidence implicating these networks in AD and other dementias.^33^ Similarly, affective dysregulation may indicate early disruption of emotional regulatory networks that precedes overt cognitive decline, potentially representing a behavioral manifestation of incipient dementia.^22^

The quantitative synthesis further reinforces these observations. Across eight independent cohorts, the pooled HR indicated that individuals with MBI had more than double the risk of developing dementia compared with those without later-life emergent behavioral symptoms (HR = 2.70, 95% CI: 2.07–3.51). Although heterogeneity was substantial, reflecting differences in assessment tools, follow-up durations, and population characteristics, the direction of effect was remarkably consistent. Evidence of funnel plot asymmetry suggests potential publication bias or true heterogeneity driven by smaller clinical samples with higher baseline risk. Nonetheless, the robustness and consistency of the pooled association support the interpretation of MBI as an early manifestation of dementia.

The predominance of AD as the most frequent dementia subtype following MBI in large registry-based and community cohorts^22,23,25,29^ suggests that behavioral symptoms often emerge prior to, or concomitantly with, pathological processes typically detected through cognitive assessment. This finding likely reflects the high population prevalence of clinically diagnosed AD, rather than a subtype-specific association between MBI and AD. In contrast, a neurology clinic-based cohort demonstrated a much higher conversion rate to behavioral variant frontotemporal dementia^31^ among 113 individuals with MBI, highlighting the influence of study context and sample characteristics. This pattern suggests that referral bias and the enrichment of specialized clinical populations may shape the distribution of dementia subtypes. Taken together, these findings indicate that MBI may represent an early clinical expression of multiple dementia subtypes, rather than a construct specific to AD. In the same line, the female predominance in the MBI studies is concordant with female sex as a risk factor for dementia, including AD.^34^

Biomarker studies provided relevant information into the pathophysiological basis of MBI. Elevated plasma p-tau181 levels, increased amyloid accumulation, and structural brain changes including reduced cortical thickness and increased white matter hyperintensities were observed among individuals with MBI.^23,24,28,31^ These findings support a mechanistic link between early behavioral changes and underlying neurodegenerative processes, indicating that MBI may serve as a clinically observable correlate of molecular and structural pathology, and potentially as a target for early intervention before significant cognitive decline becomes apparent. In this context, MBI offers a clinically accessible behavioral marker that may precede measurable cognitive impairment, expanding the window for intervention. As disease-modifying therapies begin to enter clinical practice, recognizing MBI as an early manifestation of underlying pathology could enhance patient selection for emerging treatments and improve the likelihood of therapeutic benefit. Additional pathophysiological pathways must be investigated, including TDP-43 pathology, inflammatory processes, and other potential contributors.

Importantly, the relationship between MBI and dementia is modulated by genetic, sociodemographic, and comorbid factors. The paradoxical effect of *APOE* ε4, which increases dementia risk among individuals with MBI-related apathy but attenuates risk in those with affective dysregulation,^22,29^ highlights the complex interplay between genetic susceptibility and behavioral phenotypes, suggesting that the same genetic risk factor may interact differentially with distinct neural circuits underlying specific behavioral domains. It is also tempting to speculate that other genetic factors may exert similar influences and deserve further investigation. Racial or ethnic differences, as evidenced by stronger associations between MBI and dementia among Black participants,^22^ can indicate the potential influence of social determinants of health, access to care, or population-specific biological factors. Co-occurring conditions such as sleep disturbances were also shown to amplify dementia risk in individuals with MBI,^26^ supporting a multidimensional model in which behavioral symptoms, physiological vulnerabilities, and environmental influences converge to shape neurodegenerative trajectories.

Despite the strength of the current evidence, several limitations must be considered, notably the heterogeneity of study designs, samples and clinical instruments. This heterogeneity precluded sensitivity or subgroup analyses. Most studies relied on informant-based measures such as the NPI (clinician-administered, informant-reported) or the NPI-Q (informant-completed) to operationalize MBI, which may be subject to reporting bias. Incorporating structured clinician-rated assessments or diagnostic interviews conducted directly with the patient could help mitigate this issue and provide a more reliable characterization of behavioral changes. This approach could also promote a better understanding of the interaction between MBI and psychiatric disorders, as defined by DSM-5. Of clinical relevance, it remains to be determined how MBI and its neurobehavioral domains (e.g., apathy, affective dysregulation) overlap and/or differ from psychiatric conditions, especially depression, that have been linked to AD and other dementias. The heterogeneity of assessment instruments hinders cross-study comparisons, and the more widespread adoption of the MBI-Checklist could help standardize measurement and improve comparability across studies. The length of clinical follow-up was also significantly different. Several studies did not report dementia subtypes, which limits the ability to characterize subtype-specific conversion patterns. In addition, there is the need to specifically investigate whether distinct neurobehavioral domains carry different weights in shaping outcome trajectories and risk for specific dementia subtypes. While biomarkers were assessed in a few studies, integration with longitudinal behavioral trajectories remains very limited, precluding definitive causal inferences about the temporal relationship between MBI onset and neuropathological progression. Another important limitation is the narrow scope of biomarker investigations as the studies have focused primarily on AD-related markers (e.g., amyloid and tau), overlooking inflammation, vascular, among others.^23,28^

Taken together, despite methodological heterogeneity, the evidence synthesized in this review suggests a consistent association between MBI and incident dementia. Across diverse populations, settings, and operational definitions, individuals with MBI showed more than double the risk of developing dementia. Converging biomarker findings further support the interpretation of MBI as an early neurobehavioral expression of underlying neurodegenerative processes.

These findings point to future research directions that can validate MBI as a clinically and biologically meaningful syndrome within dementia frameworks. Standardizing MBI assessment would improve comparability among studies, eventually allowing the development of domain-specific risk stratification models. Longitudinal studies integrating neurobehavioral, cognitive, genetic, and biomarker data are needed to clarify the mechanistic pathways linking MBI to dementia. Additionally, examining the influence of sociodemographic characteristics and physical comorbidities will be critical for refining risk stratification and tailoring early intervention strategies. As no previous study has examined whether treatment of MBI influences the trajectory of cognitive decline, this is a pivotal issue to be addressed. Thus, future investigations should evaluate whether different therapeutic modalities targeting MBI, including pharmacological and non-pharmacological (e.g., physical activity, dietary modification, sleep optimization, and behavioral therapies) interventions, can interfere with dementia progression. From a clinical perspective, the accumulating evidence supports consideration of systematic MBI screening in older adults, particularly among those who are cognitively normal. Such an approach may enable identification of individuals at elevated risk before the onset of overt cognitive impairment, thereby creating a potential window for early intervention, monitoring, and preventive strategies.

## Supplemental Material

sj-docx-1-alz-10.1177_13872877261480315 - Supplemental material for Mild behavioral impairment and risk of incident dementia: A systematic review and meta-analysis Supplemental material, sj-docx-1-alz-10.1177_13872877261480315 for Mild behavioral impairment and risk of incident dementia: A systematic review and meta-analysis by Karina L. M. Sassi, Anika Thomson, Vanessa M. Young, Natalia P. Rocha and Antonio L. Teixeira in Journal of Alzheimer's Disease

## Data Availability

The datasets analyzed during the current study were derived from previously published studies cited in this article. The extracted data are available from the corresponding author upon reasonable request.
