# Supplementary material for: Mild behavioral impairment and risk of incident dementia: A systematic review and meta-analysis
Source: J Alzheimers Dis. 2026 Sep 7;113(4):1587–601. doi: 10.1177/13872877261480315 (PMC13688341; doi:10.1177/13872877261480315)
Supplement: Supplementary file 1 [file sj-docx-1-alz-10.1177_13872877261480315.docx]

**Supplemental Material**

**Mild behavioral impairment and risk of incident dementia: A systematic review and meta-analysis**

**Supplemental Table 1. Search Strategies**

**PubMed**

("mild behavioral impairment"[tiab] OR "Mild Behavioral Impairment"[Mesh] OR "neuropsychiatric symptoms"[tiab] OR "Behavioral Symptoms"[Mesh] OR "behavioral and psychological symptoms"[tiab] OR "non-cognitive symptoms"[tiab]) AND (predict*[tiab] OR progress*[tiab] OR conversion[tiab] OR risk[tiab]) AND (dementia[tiab] OR "Dementia"[Mesh] OR "Alzheimer Disease"[Mesh] OR Alzheimer*[tiab] OR "frontotemporal dementia"[tiab] OR "cognitive decline"[tiab]) AND (longitudinal[tiab] OR prospective[tiab] OR "follow-up"[tiab] OR cohort[tiab] OR "Cohort Studies"[Mesh])

**Scopus**

TITLE-ABS-KEY ( "mild behavioral impairment" OR "neuropsychiatric symptoms" OR "behavioral symptoms" OR "behavioral and psychological symptoms" OR "non-cognitive symptoms" )

AND

TITLE-ABS-KEY ( predict* OR progress* OR conversion OR risk )

AND

TITLE-ABS-KEY ( dementia OR Alzheimer* OR "cognitive decline" OR "frontotemporal dementia" )

AND

TITLE-ABS-KEY ( longitudinal OR prospective OR "follow-up" OR cohort )

**Embase**

('mild behavioral impairment'/exp OR 'mild behavioral impairment':ti,ab,kw OR 'neuropsychiatric symptoms'/exp OR 'neuropsychiatric symptoms':ti,ab,kw OR 'behavior symptom'/exp OR 'behavioral symptoms':ti,ab,kw OR 'behavioral and psychological symptoms':ti,ab,kw OR 'non cognitive symptoms':ti,ab,kw) AND (predict*:ti,ab,kw OR progress*:ti,ab,kw OR conversion:ti,ab,kw OR risk:ti,ab,kw) AND ('dementia'/exp OR dementia:ti,ab,kw OR 'Alzheimer disease'/exp OR Alzheimer*:ti,ab,kw OR 'frontotemporal dementia'/exp OR 'frontotemporal dementia':ti,ab,kw OR 'cognitive decline':ti,ab,kw) AND (longitudinal:ti,ab,kw OR prospective:ti,ab,kw OR 'follow up':ti,ab,kw OR 'cohort analysis'/exp OR cohort:ti,ab,kw)

**PsycInfo**

("mild behavioral impairment") OR ("neuropsychiatric symptoms") OR ("behavioral symptoms") OR ("behavioral and psychological symptoms") OR ("non-cognitive symptoms") - in any field

AND

(predict* OR progress* OR conversion OR risk) – in any field

AND

(dementia OR "cognitive decline" OR "frontotemporal dementia" OR Alzheimer* - in any field

AND

(longitudinal OR prospective OR "follow-up") – in any field

**Scielo**

("mild behavioral impairment" OR "neuropsychiatric symptoms" OR "behavioral symptoms" OR "behavioral and psychological symptoms" OR "non-cognitive symptoms") AND (predict* OR progress* OR conversion OR risk) AND (dementia OR Alzheimer* OR "cognitive decline

**Supplemental Table 2. Excluded Studies with rationale**

| **Studies Excluded (Author, Year)** | **Summary Comment** |
| --- | --- |
| Bidzan et al., 2008 ^1^ | No exposure of interest |
| Bray et al., 2021 ^2^ | Conference Abstract |
| Burhanullah et al., 2020 ^3^ | No exposure of interest |
| Cano et al., 2018 ^4^ | Conference Abstract |
| Chen et al., 2025 ^5^ | No exposure of interest |
| Cosseddu et al., 2020 ^6^ | Population not of interest |
| Creese et al., 2019 ^7^ | No exposure of interest |
| Ferraro et al., 2021 ^8^ | Conference Abstract |
| Ferraro et al., 2023 ^9^ | No outcome of interest |
| Fieldhouse et al., 2021 ^10^ | Population not of interest |
| Fieldhouse et al., 2024 ^11^ | Population not of interest |
| Flores-Vázquez et al., 2024 ^12^ | Conference Abstract |
| Gill et al., 2019 ^13^ | Conference Abstract |
| Gosselin et al., 2022 ^14^ | No outcome of interest |
| Guan et al. 2023 ^15^ | Study design not eligible |
| Ismail et al., 2018 ^16^ | Conference Abstract |
| Ismail et al., 2019 ^17^ | Conference Abstract |
| Ismail et al., 2022 ^18^ | Conference Abstract |
| Jin et al., 2025 ^19^ | Study design not eligible |
| Kan et al., 2025 ^20^ | No outcome of interest |
| Kassam et al., 2023 ^21^ | No outcome of interest |
| Kushwaha et al., 2024 ^22^ | Conference Abstract |
| Leow et al., 2024 ^23^ | Study design not eligible |
| Li et al., 2014 ^24^ | Study design not eligible |
| Macedo et al., 2023 ^25^ | Conference Abstract |
| Mallo et al., 2023 ^26^ | Conference Abstract |
| Matsuoka et al., 2021 ^27^ | No outcome of interest |
| Miao et al., 2021 ^28^ | No outcome of interest |
| Mortby, 2021 ^29^ | Study design not eligible |
| Neergaard et al., 2017 ^30^ | No exposure of interest |
| Richey et al., 2024 ^31^ | Conference Abstract |
| Robert et al., 2008 ^32^ | No exposure of interest |
| Rykov et al., 2024 ^33^ | No outcome of interest |
| Ryu et al., 2011 ^34^ | No outcome of interest |
| Sevinçer et al., 2017 ^35^ | Population not of interest |
| Sharif et al., 2025 ^36^ | Study design not eligible |
| Sheikh et al., 2018 ^37^ | No outcome of interest |
| Scholzel-Dorenbos et al., 2006 ^38^ | Study design not eligible |
| Steinberg et al., 2015 ^39^ | Conference Abstract |
| Soto et al., 2018 ^40^ | Conference Abstract |
| Taragano et al., 2008 ^41^ | No exposure of interest |
| Taragano et al., 2009 ^42^ | No exposure of interest |
| Taragano et al., 2018 ^43^ | No exposure of interest |
| Tschanz et al., 2013 ^44^ | No exposure of interest |
| Tsunoda et al., 2021 ^45^ | No exposure of interest |
| van der Linde et al., 2013 ^46^ | No exposure of interest |
| Yokoi et al., 2019 ^47^ | No exposure of interest |
| Yoon et al., 2023 ^48^ | No exposure of interest |
| Wise et al., 2019 ^49^ | No exposure of interest |
| Wolfova et al., 2021 ^50^ | No outcome of interest |
| Wyman-Chick et al., 2018 ^51^ | Conference Abstract |

**Supplemental Table 3. Newcastle–Ottawa Scale to evaluate risk of bias**

**NEWCASTLE–OTTAWA SCALE - COHORT RISK-OF-BIAS**

| **Study** | **Representativeness** | **Non‑exposed selection** | **Ascertainment of exposure** | **Outcome not present at start** | **Comparability (criteria A and B)** | **Outcome assessment** | **Follow‑up long enough** | **Adequacy of follow‑up** | **Total** | **Risk** |
| --- | --- | --- | --- | --- | --- | --- | --- | --- | --- | --- |
| **Matsuoka et al., 2019^52^** | 🟢 | 🟢 | 🟢 | 🟢 | 🟢 🔴 | 🟢 | 🟢 | 🔴 | **7/9** | Low |
| **Orso et al., 2020^53^** | 🟢 | 🟢 | 🟢 | 🟢 | 🔴 🔴 | 🟢 | 🟢 | 🟢 | **7/9** | Low |
| **Ismail et al., 2021^54^** | 🟢 | 🟢 | 🟢 | 🟢 | 🟢 🟢 | 🟢 | 🟢 | 🟢 | **9/9** | Low |
| **Sun et al., 2021^55^** | 🟢 | 🟢 | 🟢 | 🟢 | 🟢 🟢 | 🟢 | 🟢 | 🔴 | **8/9** | Low |
| **Vellone et al., 2022^56^** | 🟢 | 🟢 | 🟢 | 🟢 | 🟢 🟢 | 🟢 | 🟢 | 🔴 | **8/9** | Low |
| **Ebrahim et al., 2023^57^** | 🟢 | 🟢 | 🟢 | 🟢 | 🟢 🟢 | 🟢 | 🟢 | 🔴 | **8/9** | Low |
| **Ghahremani et al., 2023^58^** | 🟢 | 🟢 | 🟢 | 🟢 | 🟢 🟢 | 🟢 | 🟢 | 🔴 | **8/9** | Low |
| **Mudalige et al., 2023^59^** | 🟢 | 🟢 | 🟢 | 🟢 | 🟢 🟢 | 🟢 | 🟢 | 🔴 | **8/9** | Low |
| **Gracia‑García et al., 2024^60^** | 🟢 | 🟢 | 🟢 | 🟢 | 🟢 🟢 | 🟢 | 🟢 | 🟢 | **9/9** | Low |
| **Kan et al., 2025^61^** | 🟢 | 🟢 | 🟢 | 🟢 | 🟢 🟢 | 🟢 | 🟢 | 🔴 | **8/9** | Low |
| **Richey et al., 2025^62^** | 🟢 | 🟢 | 🟢 | 🟢 | 🟢 🟢 | 🟢 | 🟢 | 🔴 | **8/9** | Low |

Color legend:
🟢 = Star awarded (low risk)
🔴 = Not met / no star awarded (high risk)

**References**

1.	Bidzan L, Pachalska M, Grochmal-Bach B, et al. Behavioral and psychological symptoms in the preclinical stage of Alzheimer's disease. *Med Sci Monit* 2008; 14: CR473–CR479.

2.	Bray MJC, Bryant BR, Esagoff AI, et al. Effect of traumatic brain injury on mild behavioral impairment domains prior to all-cause dementia diagnosis and throughout disease progression. *Alzheimers Dement (N Y)* 2022; 8: e12364.

3.	Burhanullah MH, Tschanz JT, Peters ME, et al. Neuropsychiatric symptoms as risk factors for cognitive decline in clinically normal older adults: The Cache County Study. *Am J Geriatr Psychiatry* 2020; 28: 64-71.

4.	Cano J, Chan V, Kan CN, et al. Mild behavioral impairment: prevalence in clinical setting and cognitive correlates. *Alzheimers Dement* 2018; 14: 639-640.

5.	Chen J, Numbers K, Lo J, et al. Do changed behaviors predict cognitive decline in a community sample? *Int Psychogeriatr* Epub ahead of print 28 Apr 2025: DOI: 10.1016/j.inpsyc.2025.100076.

6.	Cosseddu M, Benussi A, Gazzina S, et al. Progression of behavioural disturbances in frontotemporal dementia: a longitudinal observational study. *Eur J Neurol* 2020; 27: 265-272.

7.	Creese B, Brooker H, Ismail Z, et al. Mild behavioral impairment as a marker of cognitive decline in cognitively normal older adults. *Am J Geriatr Psychiatry* 2019; 27: 823-834.

8.	Ferraro P, De Maria E, Gervino E, et al. Exploring potential markers of pre-dementia risk states in motor neuron diseases: A longitudinal study of mild behavioral impairment and its relation to cognition. *Amyotrophic Lateral Scler Frontotemporal Degener* 2021; 22: 192.

9.	Ferraro PM, Gervino E, De Maria E, et al. Mild behavioral impairment as a potential marker of predementia risk states in motor neuron diseases. *Eur J Neurol* 2023; 30: 47-56.

10.	Fieldhouse JLP, Gossink FT, Feenstra TC, et al. Clinical phenotypes of behavioral variant frontotemporal dementia by age at onset. *J Alzheimers Dis* 2021; 82: 381-390.

11.	Fieldhouse JLP, van Engelen ME, Handgraaf D, et al. Trajectories of behavior and social cognition in behavioral variant frontotemporal dementia and primary psychiatric disorders: A call for better operationalization of socioemotional changes. *Eur J Neurol* 2024; 31: e16426.

12.	Flores-Vazquez JP, Santos-Díaz A, Marrufo-Meléndez OR, et al. Loss of fornix white-matter integrity is associated with impulse dyscontrol in cognitively normal older adults. *Int Psychogeriatr* 2024; 36 (Suppl 1): 109-110.

13.	Gill S, Wang M, Forkert ND, et al. Diffusion tensor imaging in pre-dementia risk states: white matter atrophy findings in mild behavioral impairment. *Neurology* 2019; 92 (15 Suppl): P5.1-025.

14.	Gosselin P, Guan DX, Chen HY, et al. The relationship between hearing and mild behavioral impairment and the influence of sex: a study of older adults without dementia from the COMPASS-ND Study. *J Alzheimers Dis Rep* 2022; 6: 57-66.

15.	Guan DX, Smith EE, Pike GB, et al. Persistence of neuropsychiatric symptoms and dementia prognostication: A comparison of three operational case definitions of mild behavioral impairment. *Alzheimers Dement (Amst)* 2023; 15: e12483.

16.	Ismail Z and Saykin AJ. Subjective cognitive decline (SCD) and mild behavioral impairment (MBI): the intersection of two early indicators of AD pathophysiology. *Alzheimers Dement* 2018; 14 (Suppl 7): P210-P211.

17.	Ismail Z, Naude J, Gill S, et al. Plasma neurofilament light is a marker of incident cognitive decline associated with mild behavioral impairment-lessons for clinical trial recruitment. *J Prev Alzheimers Dis* 2019; 6: 94-95.

18.	Ismail Z, Leon R, Creese B, et al. Optimizing detection of prodromal Alzheimer disease in mild cognitive impairment - a 4-year cerebrospinal fluid study of mild behavioral impairment in ADNI and MEMENTO. *J Prev Alzheimers Dis* 2022; 9 (Suppl 1): 116-117.

19.	Jin B, Yoon EJ, Woo KA, et al. Mild behavioral impairment in idiopathic REM sleep behavior disorder and Lewy body disease continuum. *J Neural Transm (Vienna)* 2025; 132: 637-644.

20.	Kan CN, Cano J, Zhao X, et al. Prevalence, clinical correlates, cognitive trajectories, and dementia risk associated with mild behavioral impairment in Asians. *J Clin Psychiatry* 2022; 83: 21m14105.

21.	Kassam F, Chen H, Nosheny RL, et al. Cognitive profile of people with mild behavioral impairment in Brain Health Registry participants. *Int Psychogeriatr* 2023; 35: 643-652.

22.	Kushwaha S, Singh M, Anthony A, et al. Baseline prevalence and longitudinal study of cognitive trajectory in Parkinson's disease. *8th Annual Conference of the Movement Disorders Society of India* 2024; 7: DOI: 10.4103/aomd.aomd_14_24.

23.	Leow YJ, Soo SA, Kumar D, et al. Mild behavioral impairment and cerebrovascular profiles are associated with early cognitive impairment in a community-based Southeast Asian Cohort. *J Alzheimers Dis* 2024; 97: 1727-1735.

24.	Li XL, Hu N, Tan MS, et al. Behavioral and psychological symptoms in Alzheimer's disease. *Biomed Res Int* 2014; 2014: 927804.

25.	Macedo AC, Tissot C, Therriaultc J, et al. PET-based Braak staging predicts neuropsychiatric burden and severity in aging and Alzheimer's disease. *J Neurol Sci* 2023; 455: 121125.

26.	Mallo SC, Lojo-Seoane C, Nieto-Vietes A, et al. Early detection and evolution of Mild Behavioral Impairment in a sample of people with subjective cognitive complaints. *Int Psychogeriatr* 2023; 35 (S1): 257.

27.	Matsuoka T, Ueno D, Ismail Z, et al. Neural correlates of mild behavioral impairment: a functional brain connectivity study using resting-state functional magnetic resonance imaging. *J Alzheimers Dis* 2021; 83: 1221-1231.

28.	Miao R, Chen HY, Robert P, et al. White matter hyperintensities and mild behavioral impairment: Findings from the MEMENTO cohort study. *Cereb Circ Cogn Behav* 2021; 2: 100028.

29.	Mortby ME. Mild behavioral impairment: challenges facing a quickly developing evidence base. *Int Psychogeriatr* 2021; 33: 209-212.

30.	Skov Neergaard J, Dragsbaek K, Christiansen C, et al. Objective cognitive impairment and progression to dementia in women: The Prospective Epidemiological Risk Factor Study. *J Prev Alzheimers Dis* 2017; 4: 194-200.

31.	Richey LN, Daneshvari NO, Young L, et al. Associations of head injury and mild behavioral impairment with cognitive function and dementia. *J Neurotrauma* 2024; 41: 15-16.

32.	Robert PH, Berr C, Volteau M, et al. Importance of lack of interest in patients with mild cognitive impairment. *Am J Geriatr Psychiatry* 2008; 16: 770-776.

33.	Rykov YG, Ng KP, Patterson MD, et al. Predicting the severity of mood and neuropsychiatric symptoms from digital biomarkers using wearable physiological data and deep learning. *Comput Biol Med* 2024; 180: 108959.

34.	Ryu SH, Ha JH, Park DH, et al. Persistence of neuropsychiatric symptoms over six months in mild cognitive impairment in community-dwelling Korean elderly. *Int Psychogeriatr* 2011; 23: 214-220.

35.	Sevinçer GM, İpekçioglu DY, Konuk N, et al. The relationship between the premorbid personality traits and the behavioral and psychological symptoms of Alzheimer disease. *Neurol Psychiatry Brain Res* 2017; 24: 20-25.

36.	Sharif SF, Guan DX, Bodnar TS, et al. Neuropsychiatric symptoms and progression to pathologically confirmed Alzheimer's disease. *Brain* 2025; 148: 3694-3704.

37.	Sheikh F, Ismail Z, Mortby ME, et al. Prevalence of mild behavioral impairment in mild cognitive impairment and subjective cognitive decline, and its association with caregiver burden. *Int Psychogeriatr* 2018; 30: 233-244.

38.	Scholzel-Dorenbos CJ. Mild behavioral impairment: a prodromal stage of frontotemporal lobar degeneration. *J Am Geriatr Soc* 2006; 54: 180-181.

39.	Steinberg MI and Leoutsakos JS. The association between general medical health and course of cognition, function, and neuropsychiatric symptoms in the cache county dementia progression study. *Alzheimers Dement* 2015; 11 (Suppl): P261.

40.	Soto M, Simon D, Kiyasova V, et al. Neuropsychiatric symptoms and the risk of conversion to dementia due to Alzheimer disease among MCI subjects. *Alzheimers Dement* 2018; 14 (Suppl): P1198.

41.	Taragano FE, Allegri RF and Lyketsos C. Mild behavioral impairment: A prodromal stage of dementia. *Dement Neuropsychol* 2008; 2: 256-260.

42.	Taragano FE, Allegri RF, Krupitzki H, et al. Mild behavioral impairment and risk of dementia: a prospective cohort study of 358 patients. *J Clin Psychiatry* 2009; 70: 584-592.

43.	Taragano FE, Allegri RF, Heisecke SL, et al. Risk of conversion to dementia in a mild behavioral impairment group compared to a psychiatric group and to a mild cognitive impairment group. *J Alzheimers Dis* 2018; 62: 227-238.

44.	Tschanz JT, Norton MC, Zandi PP, et al. The Cache County Study on Memory in Aging: factors affecting risk of Alzheimer's disease and its progression after onset. *Int Rev Psychiatry* 2013; 25: 673-685.

45.	Tsunoda K, Yamashita T, Osakada Y, et al. Positive baseline behavioral and psychological symptoms of dementia predict a subsequent cognitive impairment in cognitively normal population. *Neurol Clin Neurosci* 2021; 9: 218-222.

46.	van der Linde RM, Stephan BC, Matthews FE, et al. The presence of behavioural and psychological symptoms and progression to dementia in the cognitively impaired older population. *Int J Geriatr Psychiatry* 2013; 28: 700-709.

47.	Yokoi Y, Takano H, Sakata M, et al. Discrete effect of each mild behavioural impairment category on dementia conversion or cognitive decline in patients with mild cognitive impairment. *Psychogeriatrics* 2019; 19: 591-600.

48.	Yoon EJ, Lee JY, Kwak S, et al. Mild behavioral impairment linked to progression to Alzheimer's disease and cortical thinning in amnestic mild cognitive impairment. *Front Aging Neurosci* 2022; 14: 1051621.

49.	Wise EA, Rosenberg PB, Lyketsos CG, et al. Time course of neuropsychiatric symptoms and cognitive diagnosis in National Alzheimer's Coordinating Centers volunteers. *Alzheimers Dement (Amst)* 2019; 11: 333-339.

50.	Wolfova K, Creese B, Aarsland D, et al. Gender/sex differences in the association of mild behavioral impairment with cognitive aging. *J Alzheimers Dis* 2022; 88: 345-355.

51.	Wyman-Chick KA, Erickson LO, Rosenbloom MH, et al. Prevalence of neuropsychiatric and motor symptoms in prodromal dementia with Lewy bodies. *Alzheimers Dement* 2018; 14 (Suppl): P1645-P1646.

52.	Matsuoka T, Ismail Z and Narumoto J. Prevalence of mild behavioral impairment and risk of dementia in a psychiatric outpatient clinic. *J Alzheimers Dis* 2019; 70: 505-513.

53.	Orso B, Mattei C, Arnaldi D, et al. Clinical and MRI predictors of conversion from mild behavioural impairment to dementia. *Am J Geriatr Psychiatry* 2020; 28: 755-763.

54.	Ismail Z, McGirr A, Gill S, et al. Mild behavioral impairment and subjective cognitive decline predict cognitive and functional decline. *J Alzheimers Dis* 2021; 80: 459-469.

55.	Sun Y, Xu W, Chen KL, et al. Mild behavioral impairment correlates of cognitive impairments in older adults without dementia: mediation by amyloid pathology. *Transl Psychiatry* 2021; 11: 577.

56.	Vellone D, Ghahremani M, Goodarzi Z, et al. Apathy and APOE in mild behavioral impairment, and risk for incident dementia. *Alzheimers Dement (N Y)* 2022; 8: e12370.

57.	Ebrahim IM, Ghahremani M, Camicioli R, et al. Effects of race, baseline cognition, and APOE on the association of affective dysregulation with incident dementia: A longitudinal study of dementia-free older adults. *J Affect Disord* 2023; 332: 9-18.

58.	Ghahremani M, Wang M, Chen HY, et al. Plasma phosphorylated tau at threonine 181 and neuropsychiatric symptoms in preclinical and prodromal Alzheimer disease. *Neurology* 2023; 100: e683-e693.

59.	Mudalige D, Guan DX, Ghahremani M, et al. Longitudinal associations between mild behavioral impairment, sleep disturbance, and progression to dementia. *J Alzheimers Dis Rep* 2023; 7: 1323-1334.

60.	Gracia-Garcia P, Lopez-Anton R, de la Camara C, et al. Mild behavioral impairment in the general population aged 55+ and its association with incident dementia. *Alzheimers Dement (Amst)* 2024; 16: e12610.

61.	Kan CN, Hilal S, Xu X, et al. Comorbid cerebrovascular and neurodegenerative burden in mild behavioural impairment and their impact on clinical trajectory. *Acta Neuropsychiatr* 2025; 37: e53.

62.	Richey LN, Daneshvari NO, Young L, et al. Associations of traumatic brain injury and mild behavioral impairment with cognitive function and dementia. *J Geriatr Psychiatry Neurol* 2025; 38: 264-274.
